# Treatment of glioblastoma multiforme with “classic” 4:1 ketogenic diet total meal replacement

**DOI:** 10.1186/s40170-020-00230-9

**Published:** 2020-11-09

**Authors:** Pavel Klein, Ivana Tyrlikova, Giulio Zuccoli, Adam Tyrlik, Joseph C. Maroon

**Affiliations:** 1 Mid-Atlantic Epilepsy and Sleep Center, 6410 Rockledge Drive, Suite 610, Bethesda, MD 20817 USA; 2 Program for the Study of Neurodevelopment in Rare Disorders (NDRD), University of Pittsburgh Medical Center, Pittsburgh, PA 15213 USA; 3 Department of Neurosurgery, University of Pittsburgh Medical Center, Pittsburgh, PA 15213 USA

**Keywords:** Glioblastoma multiforme, Ketogenic diet, Low carb diet

## Abstract

**Introduction:**

Glioblastoma (GBM) has poor survival with standard treatment. Experimental data suggest potential for metabolic treatment with low carbohydrate ketogenic diet (KD). Few human studies of KD in GBM have been done, limited by difficulty and variability of the diet, compliance, and feasibility issues. We have developed a novel KD approach of total meal replacement (TMR) program using standardized recipes with ready-made meals. This pilot study evaluated feasibility, safety, tolerability, and efficacy of GBM treatment using TMR program with “classic” 4:1 KD.

**Method:**

GBM patients were treated in an open-label study for 6 months with 4:1 [fat]:[protein + carbohydrate] ratio by weight, 10 g CH/day, 1600 kcal/day TMR. Patients were either newly diagnosed (group 1) and treated adjunctively to radiation and temozolomide or had recurrent GBM (group 2). Patients checked blood glucose and blood and urine ketone levels twice daily and had regular MRIs. Primary outcome measures included retention, treatment-emergent adverse events (TEAEs), and TEAE-related discontinuation. Secondary outcome measures were survival time from treatment initiation and time to MRI progression.

**Results:**

Recruitment was slow, resulting in early termination of the study. Eight patients participated, 4 in group 1 and 4 in group 2. Five (62.5%) subjects completed the 6 months of treatment, 4/4 subjects in group 1 and 1/4 in group 2. Three subjects stopped KD early: 2 (25%) because of GBM progression and one (12.5%) because of diet restrictiveness. Four subjects, all group 1, continued KD on their own, three until shortly before death, for total of 26, 19.3, and 7 months, one ongoing. The diet was well tolerated. TEAEs, all mild and transient, included weight loss and hunger (*n* = 6) which resolved with caloric increase, nausea (*n* = 2), dizziness (*n* = 2), fatigue, and constipation (*n* = 1 each). No one discontinued KD because of TEAEs. Seven patients died. For these, mean (range) survival time from diet initiation was 20 months for group 1 (9.5–27) and 12.8 months for group 2 (6.3–19.9). Mean survival time from diagnosis was 21.8 months for group 1 (11–29.2) and 25.4 months for group 2 ( 13.9–38.7). One patient with recurrent GBM and progression on bevacizumab experienced a remarkable symptom reversal, tumor shrinkage, and edema resolution 6–8 weeks after KD initiation and survival for 20 months after starting KD.

**Conclusion:**

Treatment of GBM patients with 4:1 KD using total meal replacement program with standardized recipes was well tolerated. The small sample size limits efficacy conclusions.

**Trial registration:**

NCT01865162 registered 30 May 2013, and NCT02302235 registered 26 November 2014, https://clinicaltrials.gov/

## Introduction

Malignant gliomas are the commonest brain tumor in adults, with approximately 12,000 new cases annually in the USA [1, 2]. Standard therapy for glioblastoma multiforme (GBM) includes maximal feasible resection followed by radiation and chemotherapy, with bevacizumab rescue therapy for recurrence. Median survival after diagnosis is approximately 15 months [3–5]. Essentially all patients suffer recurrent disease, usually within 8 months of diagnosis. The median survival after recurrence is 25 weeks with the standard treatment of bevacizumab, with a 6-month progression-free survival of 15% [5]. There is thus need for new treatment.

In the last 10 years, there has been a growing interest in alternative, metabolic treatments of GBM [6–13]. GBM cancer cells utilize aerobic fermentation of glucose in the cytosol for energy supply instead of mitochondrial oxidative phosphorylation (the “Warburg effect”) [7, 8, 10]. 18 F-fluoro-2-deoxyglucose positron emission tomography (PET) shows that human GBMs have much higher glucose utilization than normal cortex. In states of prolonged glucose deprivation, such as fasting or starvation, normal brain cells metabolize ketone bodies derived from fatty acids for energy instead of glucose. Tumor cells are poorly able to do so. They depend on glucose and glycolysis for survival. This makes tumor cells vulnerable to therapies of glucose restriction [6, 8, 10–15].

Glucose reduction and ketone exposure reduce proliferation and growth rate of human GBM cells [16] and of rodent astrocytoma lines [14]. The effect occurs independently with both glucose reduction and with ketone body exposure, with a synergistic effect between the two. Glucose deprivation results in apoptotic death in human GBM cells but not in normal cells [16]. Similarly, ketone bodies inhibit the viability of cultured human GBM cells, but not of normal cells. In mice, high circulating glucose levels accelerate tumor growth and angiogenesis and prevent apoptosis [17, 18]. Reduction in circulating glucose and increase in ketone levels through ketogenic diet (KD) with caloric restriction have pro-apoptotic, anti-angiogenic, and anti-inflammatory effects, reduce expression of mTOR effector in mice with experimental malignant gliomas, reduce rodent tumor growth and tumor size, and increase survival of animals [15–17, 19–21]. In a mouse model of malignant glioma treatment with 4:1 KD, KD increased median survival by 22% from 23 to 28 days [22]. Remarkably, treatment with KD given together with radiation resulted in complete tumor remission in 9/11 animals. KD treatment lasted for 101 days. Animals remained tumor-free until they were sacrificed at 299 days [22]. Calorically restricted KD combined with the glutamine antagonist, 6-diazo-5-oxo-L-norleucine (DON), appears similarly effective in 2 mouse models of orthoptic implanted GBM [21].

In patients with GBM, hyperglycemia is associated with shorter survival [23]. There have been seven reports of KD treatment of patients with GBM: 3 of 1–2 patients and 4 with 9–20 patients [24–30]. Outcomes in the anecdotal cases were better than expected [24, 25, 29]. However, in the larger studies, no positive findings were reported [26–28, 30, 31]. These studies have varied widely. They have ranged in treatment duration from 6–14 weeks, in disease stage from adjunctive treatment with standard initial care to recurrent GBM, in diet CH content from 60 g CH/day or 25% of calories derived from CH to 4:1 KD [26–28, 30], in fat content, and in presence, absence, and degree of caloric restriction.

Studies with > 2 patients reported difficulty with doing the diet and, in 3/4 studies, continuing it beyond 3 months. In these 3 studies, the diet duration was limited to 6 weeks–3 months because the investigators thought that patients could not tolerate longer periods. In one study, treatment continued for 14 weeks, but increased CH content after 8 weeks and required “intense counseling” [30]. There are three main problems with the “classic” 3:1 or 4:1 ketogenic diet: (1) it is complicated to do; (2) it is not palatable; and (3) it is done individually by each patient, and thus differs between patients in a study, and between studies.

To address these challenges, we developed a novel program of total meal replacement (TMR) of ready-made 4:1 and 3:1 KD meals to simplify and standardize the diet in order to make it easier to do and adhere to, and to make it uniform across a study and comparable between studies. The program delivers patients ready-made meals using a large palette of our own recipes in a TMR program with 4:1 KD with 10 g CH and 1600 kcal/day, with no other food consumed. The goal of this pilot study was to evaluate feasibility, safety, and tolerability of GBM treatment with this program and to obtain pilot data on efficacy.

## Methods

This was a prospective open label study. The initial protocol was for treatment of recurrent “terminal” GBM after resection, radiation, temozolomide, and failed rescue therapy with bevacizumab. Because of good tolerance and good response of the first subject, a second study was started for adjunctive KD treatment early in the disease, concomitant with initial radiation and temozolomide therapies. The evaluation and treatment protocols of both studies were identical. Because of slow recruitment into both studies, we report the combined treatment of both protocols. Both protocols were approved by the institutional review board of Holy Cross Hospital, Silver Spring, MD. All subjects signed IRB-approved consent form. The study was conducted at the Mid-Atlantic Epilepsy and Sleep Center, Bethesda MD, and was registered as NCT01865162 (recurrent GBM) and NCT02302235 (newly diagnosed GBM).

### Study design

This was an open label phase 1 study treatment of adults with GBM for 6 months with 4:1 [fat]:[protein + carbohydrate] ratio, 1600 kcal/day diet. Treatment was initiated either early in the disease, with initiation of radiation and temozolomide therapy (group 1) or following recurrence (group 2). Inclusion criteria, other than disease stage, evaluations, and treatment protocols were identical for both groups. Primary outcomes were feasibility, safety, and tolerability; secondary outcome was efficacy. Primary outcome measures included, for feasibility, (1) retention in the study; for safety, (2) treatment emergent adverse events (TEAEs); and (3) treatment discontinuation because of TEAEs. Secondary outcome measures were, for efficacy, (4) overall survival time from treatment initiation and (5) time to MRI progression. Other outcome measures included treatment compliance, hunger scale scores, fasting serum glucose, and beta-hydroxy butyrate (BHB) levels and urine ketone levels.

### Subjects

Subjects were men and women aged 18–65 with histologically confirmed GBM of either early stage (after initial surgery/biopsy) or late stage (recurrence or progression after radiation and temozolomide treatment). Patients in group 2 had to have measurable contrast-enhancing progressive or recurrent GBM by MRI imaging. Exclusion criteria included Karnofsky Performance Score < 70, anticoagulation treatment with coumadin ≥ 1 mg/day, history of non-glioma malignancy within 2 years, history of uncontrolled hyperlipidemia, renal calculi, hyperuricemia, mitochondrial disease, disorders of fatty acid metabolism, porphyria, carnitine deficiency, pancreatitis, and presence of any other unstable illness.

### Evaluations

#### Screening

Pathology of the GBM was confirmed by neuropathology review. Patients with recurrent GBM had to have MRI-documented tumor progression or recurrence. Baseline laboratory studies included serum electrolytes, renal and liver functions, CBC, PT/PTT, fasting blood glucose (FPG), and serum lipid profile (cholesterol, triglycerides, high-, low-density lipoprotein, [HDL, LDL]), uric acid levels, and serum BHB*.*

The subject’s known food allergies and special (e.g., religious) dietary requirements were reviewed. Participants were taught to measure urine ketone body (KB) levels using Ketostix (Bayer AG, Germany) which measures acetoacetate, and blood for glucose and ketone levels using self-administered Precision Xtra® Meter (Abbot Diabetes Care, Alameda, CA, USA) which measures BHB. These were done fasted in the morning and 2 h post-prandially in the evening. Subjects were instructed to keep urine ketone/blood glucose and ketone diary.

#### Subsequent evaluations

Subsequent evaluations included face-to-face visits on treatment days 7, 14, and 28 to review possible early AEs, and for further education about the diet, then monthly for the 6 months of treatment and post-treatment months 6–12, and quarterly afterward, the latter either face-to-face or by telephone. With 2 subjects, some visits occurred via Skype because of the subjects’ long distance from the site. During each visit AEs, Karnofsky Performance Score, treatment compliance, issues with KD, urine ketone body, and blood ketone and glucose diaries were reviewed. Hunger was evaluated using 7-point Likert scale (no hunger–extremely hungry). For patients with > 5% BMI loss, caloric restriction was stopped. Caloric supplementation required to remain weight-neutral was calculated, with instructions to add extra calories using 100% fat-containing calories such as olive oil or 100% fat dairy produce.

Baseline laboratory evaluations were repeated at treatment months 1, 2, 3, 4, and 6. Blood was drawn at 8 am, following an 8 h fast. MRI of the brain was performed before starting treatment (baseline) and at treatment months 2, 4, and 6, and every 2–3 months after that.

### Treatment diet

KD consisted of 4:1 [fat]:[protein + carbohydrate] ratio by weight, with 10 g CH/day, and with 1600 kcal restriction. We chose 4:1 KD because the animal KD study with the greatest treatment effect to date used 4:1 KD [22]. Patients who did not tolerate the 4:1 ratio could choose 3:1 ratio with 20 g CH/day. The diet was supplemented with vitamins, calcium, and phosphorus supplements to meet the requirements of US Dietary Reference Intakes (DRI) standard. The program consisted of 5 meals/day (breakfast, morning snack, lunch, afternoon snack, dinner), different for each day of a 2-week cycle, with repeating cycles. All meals were prepared using designed recipes (Anemone LLC, Bethesda, MD). All participants received the same meal plan but with recipe adaptation to allow personal or religious dietary restrictions (vegetarian, *n* = 1, no pork, *n* = 1) with the same caloric and macronutrient composition. Meals were prepared uniformly by one catering facility and were delivered frozen once a week. Participants were counseled not to eat any other food or and drink only 0 calorie beverages. One patient administered the diet on his own after the first 2 months, using the same KD parameters. The food was provided free by Anemone LLC.

### Medication adjustment

For subjects on steroids (*n* = 5), attempts were made to taper off steroids as quickly as clinically feasible.

### Compliance

Compliance with the diet was evaluated at each visit by reviewing patients’ consumption of food supplied by the study, extra food consumed instead of or in addition to study food, subjects’ urine and blood ketone diaries, and monthly serum β-hydroxybutyrate levels. It was scored as a composite of these factors on a 0–3 scale as 3 = complete compliance, 2 = partial, substantial compliance, 1 = partial, slight compliance, and 0 = complete non-compliance.

### Statistical analysis

Only descriptive statistics were used because of the small sample size.

## Results

### Enrollment, subject disposition, and feasibility

Eight subjects were enrolled between June 2014 and April 2019 (2 women, mean age 49.8 years (range 40–64) (Table 1). The study planned to enroll 6 subjects with recurrent GBM and 20 subjects with newly diagnosed GBM. Recruitment was slow resulting in early study termination. Six subjects were self-referred via the web (clinicaltrials.gov); 1 each was referred by a neurosurgeon and by an oncologist. Additional 27 eligible subjects declined participation after screening. Ratio of eligible screened: enrolled patients was 3.37. Reasons for non-participation after screening included discouragement by treating oncologists (*n* = 11), unwillingness to undergo food restriction (*n* = 8) or alcohol abstinence (*n* = 2), and distance from the site (*n* = 6). Of the 8 subject who enrolled, 5 were either young with young children (*n* = 3) and determined to search for all treatments possible or were determined to do everything possible to fight the disease (*n* = 2). Six had extremely supportive spouses (*n* = 5) or children (*n* = 1) who shared or, in 2 cases, initiated, interest in and search for adjunctive alternative treatments.

**Table 1:** Demographics and disease characteristics

Gender (M, %)		6 (75)
Age, years	40s	*n* = 5
	60s	*n* = 3
Race	Caucasian/white	5
	Caucasian/Hispanic	1
	Asian Indian	2
Group^1^	Group 1	4
	Group 2	4
GBM primary/secondary	Primary	6
	Secondary	2 (both in group 2)
IDH 1^2^ mutational status	Negative	6
	Unknown	2
MGMT^3^ promoter methylation status	Negative	5
	Positive	2
	Unknown	1

^1^Group 1, newly diagnosed GBM, KD concurrent with radiation and temozolomide; group 2, recurrent GBM

^2^*IDH* isocitrate dehydrogenase

^3^*MGMT* O6-methylguanine methyltransferase promoter methylation

Subject demographics and disease characteristics are shown in Table 1. Four subjects had newly diagnosed GBM (group 1); 4 had recurrent GBM (group 2). All patients in group 1 had primary GBM; 2/4 patients in group 2 had primary GBM, 2/4 had secondary evolution of grade 3 astrocytoma to GBM. Five subjects completed the 6 months of treatment per protocol (PP), 4/4 subjects in group 1 and 1/4 in group 2. Three subjects discontinued the study early: 2 (25%) due to disease progression, at 0.2 and 5 months; and 1 (12.5%) because of diet restrictiveness, at 1 month. Four subjects, all newly diagnosed, chose to continue KD on their own after completion of the 6 months’ protocol treatment. Three of them continued KD until shortly before death, for a total of 26, 19.3, and 7 months. All deceased patients were followed until death. One patient is still alive and continuing with the diet for a total of 8 months to date.

### Safety and tolerability

KD was well tolerated. No patient discontinued the diet because of TEAEs. All six patients treated for > 1 month experienced weight loss, ranging from 11–18 lb (BMI reduction range 1.9–2 kg/m^2^) and asked to stop caloric restriction. Two were overweight at diet initiation; three had normal BMI. Six patients experienced hunger which abated with caloric increase. Three patients had transient nausea, in two cases associated with BCNU therapy. Two patients had transient dizziness, and one patient each had fatigue and constipation. All TEAEs were mild to moderate. There were no treatment-emergent SAEs. Seven patients died, all of GBM. Four patients complained about the restrictiveness of the diet, including 1 who stopped the diet early and three who completed the 6 months of treatment, including two who continued the diet on their own after completion of the 6 months’ study protocol.

The commonest complaints were hunger and weight loss, leading to lifting of caloric restriction. Generally, patients liked the meal replacement program and found it palatable, with individual preferences, e.g., dislike of seafood by 2 patients. All patients commented that an a la carte choice of being able to put together daily meal programs from the TMR menu would have been preferable to the fixed daily menu program. One patient, an Amish farmer, chose to prepare his own dishes using study recipes but food ingredients from his own farm.

The 4 subjects who continued KD on their own after completion of the 6 months’ protocol treatment followed similar recipes to those used during the protocol treatment, in consultation with the study team, but with relaxation of KD ratio to 3:1, and corresponding increase in protein and carbohydrate intake (CH up to 20 g/day).

### Labs

Serum ketone, glucose and lipid levels, and urine ketone levels are shown in Tables 2 and 3. Cholesterol and triglyceride levels increased by > 20% in 3 patients each and declined by > 20% in one patient. HDL levels increased by > 20% in one patient. LDL levels increased and declined by > 20% in 2 patients each. Changes in remaining patients were slight in either direction.

**Table 2:** Mean serum beta-hydroxy butyrate (BHB) (monthly), urine ketone levels (daily), and fasting plasma glucose (FPG, daily)

Subject no.	Group no.	Serum BHB (mmol/l)		Urine ketone levels mg/dL		FPG (mg/dL)	
		Baseline	KD mean (min, max) ^1^	Baseline	KD mean (min, max)^1^	Baseline	KD mean (min, max) ^1^
1	1	2.97^2^	2.51 (1.3–4.6)	80	58.6 (15–160)	76	76.91 (49–163)
2 ^3^	1	NA^4^	2.33 (1–3)	0	68.23 (0–80)	103^3^	101.35 (77–162)
3	1	0.07	3.66 (1.9–5.3)	0	64.3 (15–160)	73	85.82 (67–112)
4	1	0.95	3.14 (1.5–6.54)	0	63.66 (5–160)	80	79.86 (64–98)
5	2	NA	0.28 (0.1–0.5)	0	4.7 (0–15)	82	95.90 (73–108)
6	2	0.36	1.08 (0.06–2.11)	0	37.92 (5–80)	106	105.46 (44–174)
7	2	0.14	NA	0	NA	59	NA
8	2	NA	0.26 (0.08–0.4)	0	24.69 (5–40)	117	75.15 (65–94)

All figures are given to the nearest two decimal points

^1^Minimum and maximum values during the whole study

^2^Patient had started KD previously on his own. BHOB was collected after screening

^3^Patient had type 2 diabetes on oral hypoglycemic medications Metformin 1500 mg/day, sulfonylurea 40 mg/day. He stopped all diabetic medications by KD week 3

^4^*NA* not available

**Table 3:** Fasting lipid profiles, baseline, and end of KD treatment

Subj. no.	Group no.	Cholesterol total			TG			HDL			LDL		
		Baseline	KD	% change	Baseline	KD	% change	Baseline	KD	% change	Baseline	KD	% change
1	1	207	242	17	64	61	− 5	55	75	36	140	155	11
2	1	128	313	145	82	136	66		82	NA		200	NA
3	1	303	282	− 7	146	128	− 12	41	48	17	232	207	− 11
4	1	188	229	22	34	38	12	107	89	− 17	74	132	78
5	2	306	232	− 24	67	33	− 51	119	101	− 15	174	124	− 29
6	2	257	235	− 9	122	251	106	92	96	4	141	89	− 37
7	2	178		NA	76		NA	45		NA	118		NA
8	2	188	225	20	108	177	64	95	91	− 4	71	99	39

### Efficacy

Seven subjects died. One subject is alive, completed the 6 months’ treatment protocol, and is continuing with the diet on her own. Only the 7 deceased subjects are included in efficacy analysis. KD treatment duration, times from diagnosis to KD initiation and to death, from treatment initiation to death and to MRI progression, and from treatment end to death are shown in Table 4. Mean survival time from diet initiation to death was 20 months for group 1 (range 9.5–27) and 12.8 months for group 2 (range 6.3–19.9 months). Mean survival from the time of diagnosis till death was 21.8 months for group 1 (range 11–29.2) and 25.4 for group 2 (range 13.9–38.7). The unusually long survival in group 2 is mainly due to two patients who had secondary GBM developing from grade 3 astrocytoma, whose survival times from GBM diagnosis were 19 and 38.7 months. Mean time from KD initiation to MRI progression was 3.4 months for group 1 (range 1.6–7.1 months), and 3.9 months for group 2 (range 0.6–9.1). One patient with recurrent GBM who had failed bevacizumab and 3 other experimental protocols before KD had tumor progression 1 week after starting KD and stopped treatment. The number of subjects was too small to analyze survival parameters by age or gene typing.

**Table 4:** Survival and progression-free survival from diagnosis, and parameters of KD initiation, KD duration during and after protocol treatment, time from KD start and KD end till death (all in months) and KD compliance

Subject no.	Group no.	Age	Overall survival^1^	Dx-KD start	Study RX duration	KD continued after study	KD start-death	KD start-MRI progression	Compliance^2^
1	1	62	29.2	2.2	6	20	27	7.8	3
2	1	64	11	1.5	6	1	9.5	1.6	3
3	1	40	25.1	1.7	6	13.3	23.4	3.9	3
5^3^	2	48	19	1.5	1	0	17.5	7.1	0
6	2	62	38.7	32.4	5	0	6.3	2.2	2
7	2	41	13.9	6.4	0.2	0	7.5	0.6	NA
8	2	41	30.1	10.2	6	0	19.9	3.8	2

Only 7 patients who died are included in the survival analysis. Group 1, newly diagnosed GBM, with KD adjunctive treatment to XRT and temozolomide. Group 2, recurrent GBM, with recurrence/progression after XRT, temozolomide, and recurrence/continued progression on bevacizumab. All months are given to the nearest decimal point

^1^Overall survival = survival from diagnosis

^2^Compliance is rated on a scale of 0–3: 0 = none, 1 = partial-slight, 2 = partial-substantial, 3 = complete

^3^Study subject no. 4 was alive at the time of submission and was therefore not included in the survival analysis

### Steroids

Five subjects were on steroids at the time of KD initiation. Two, both newly diagnosed, stopped it the day of KD initiation. They had no edema off steroids during radiation and temozolomide treatment. One patient with recurrent GBM and prominent edema tapered off dexamethasone from 8 mg within 3 weeks of starting KD, then re-started it at 2 mg/day at the insistence of her oncologist. Edema resolved during the taper. One subject continued with 2 mg dexamethasone throughout the treatment at the advice of the treating oncologist. One subject stopped dexamethasone with KD initiation, had tumor progression 2 months later, and restarted dexamethasone.

### Compliance

Compliance was complete (3 on a scale of 0–3) throughout the study in 4/8 patients; partial, substantial (2/3) in 1 patient throughout the study and in one patient for 3 months, after which it became partial slight (1/3); slight (1/3) in 1 patient; and not evaluable in one patient who stopped the study after 1 week because of disease progression. Most non-compliance consisted of extra food intake. This consisted largely of nuts; extra diary produce such as cheese, cream, and butter; low carbohydrate vegetables such as broccoli, cauliflower, and celery; and olive oil and salad dressings.

### Case reports

One subject each with new onset and recurrent GBM groups had unusually positive courses:

### Case 1 (subject no. 8, Table 4)

A 41-year old woman presented with acute obtundation and vomiting due to left temporo-parietal GBM. She underwent gross total resection, radiation, and temozolomide (Stupp protocol followed by 4 cycles of temozolamide, with thrombocytopenia). The tumor (IDH1 negative; MGMT status unknown) recurred 8 months after diagnosis, with somnolence, dysphasia, memory impairment, right homonymous hemianopsia, right-sided body neglect, 4/5 right hemiparesis, increased radiographic size on MRI of the original tumor, and a new adjacent satellite lesion with surrounding edema. Bevacizumab was started together with dexamethasone. Symptoms and MRI lesions progressed. She started 4:1 10 g CH-, 1600 kcal/day diet 10.2 months after initial presentation and continued with bevacizumab. She reduced dexamethasone from 8 to 2 mg/day without clinical or radiological change. Six weeks after diet initiation, her symptoms began to improve and resolved almost completely by 8 weeks, except for right upper quadrantanopsia. MRI showed improvement of edema and reduction of tumor size. Four months after KD initiation, MRI showed a small, clinically asymptomatic increase in lesion size compared to 1 month earlier, although the lesion was still smaller than at KD initiation. BCNU was added to bevacizumab and KD. MRI lesion size stabilized. She completed 6 months of KD and elected not to continue it due to dietary restriction (she was Asian Indian vegetarian). She was asymptomatic and radiologically stable on continued BCNU and bevacizumab for 11 months after stopping the diet. She then developed a new lesion, refused further treatment, and died 3 months later, 20 months after starting and 14 months after stopping KD.

### Case 2 (subject no. 1, Table 4)

A 62-year old previously healthy man started the diet 2 months after MRI diagnosis of right temporal GBM, with 95% resection (IDH1 status unknown; MGMT negative). KD was started with radiation and temozolomide (6 cycles). He was strictly adherent with the diet. He elected to continue with self-administered 4:1 KD with 20 g CH restriction after completing the 6 months of study treatment. He had MRI progression 7.7 months after KD initiation but remained clinically asymptomatic. He continued with KD (strictly adherent) as well as various supplements including vitamin D3, Centrum vitamins and Avemar (fermented wheat germ extract). He remained clinically asymptomatic with excellent quality of life until 2 months prior to his death when he developed pulmonary emboliand and stopped all treatment. He died 27 months after KD initiation, 29.2 months from diagnosis.

## Discussion

The main finding of this pilot study is that prolonged (6 months) treatment of patients with GBM with a rigorous, 4:1 ketogenic diet is well tolerated when delivered as a total meal replacement program using standardized recipes and ready-made meals. To our knowledge, this is the longest KD treatment study of GBM patients to date, outside of two isolated cases [25, 29]. It is the second study, other than one case report, to offer the most restrictive, “classic” 4:1 KD, which was used in our study for 26 weeks, 18 weeks longer than previously [30]. It is the first cancer ketogenic diet study in which all food intake was standardized and the same for all subjects.

### Feasibility

While preclinical data suggests great promise for KD treatment of GBM, few clinical studies have been done [24–31]. Part of the reason is the difficulty of doing KD. KD poses three main challenges: lack of palatability, the complicated nature of meal preparation, and lack of standardization. Of the four published KD GBM studies with > 2 patients, three concluded that KD cannot be tolerated for more than 6–12 weeks [26–28], and that the maximally tolerated diet is 3:1 with 20 g CH. In a German study of restrictive calorie ketogenic diet in end-stage malignant tumors, only 5/16 subjects completed the 3-month treatment period [32]. The very high fat 4:1 KD is the most difficult ketogenic diet. Recently, 9 newly diagnosed GBM patients were treated with 4:1 liquid formula KD (without caloric restriction) for 8 weeks. They tolerated it well, but after 8 weeks, the diet was liberalized to 1.5–2:0 [fat]: [CH + protein] ratio for the 6 remaining weeks of the 14 weeks of treatment [30]. Even so, the patients required “intense counseling” to execute the diet. Evaluation of results of KD treatment is difficult because the diet as currently practiced is individualized by each patient, making compliance evaluation and comparisons across patients within a study and between studies difficult.

In the present pilot study, we overcame these difficulties by providing the diet as a complete ready-made meal delivery program which was the same for all subjects (with adaptation for religious preferences and food allergies). This novel approach offers standardized treatment for all subjects, simplicity of execution for patients, and ease of monitoring compliance (patients either only eat food delivered, or add/substitute for it). We used the most challenging of ketogenic diets, the 4:1 10 g CH/day variant in which 90% of calories are derived from fat. The study shows that such an approach is feasible. Five of eight subjects completed the 6 months of treatment per protocol, 2 discontinued it because of tumor progression, and only 1 (12.5%) because of diet restrictiveness. Four subjects continued KD after completing the study protocol, for up to 26 months. This suggests that the ready-made TMR version of the diet may be easier for patients to do and adhere to. In future studies, 3:1 diet version may be offered to patients who do not tolerate the 4:1 version.

### Safety

The diet was well tolerated. No subject discontinued because of TEAEs. The main side effects were weight loss and hunger. The diet was calorie-restricted to 1600 kcal/day, but this restriction was abandoned in all 6 subjects treated for > 1 month because of weight loss. Hunger resolved with caloric increase. All other side effects, nausea, dizziness, fatigue and constipation, were mild and transient. Blood lipids were affected. Cholesterol and triglyceride levels increased markedly in two and three patients, respectively; HDL levels increased notably in 1 patient.

### Efficacy

The small study sample precludes conclusions about KD efficacy.

### Comparison with prior studies and treatment duration

Seven previous studies of KD treatment of gliomas have been reported. Three of them were 1–2 patient case reports, all showing promising results [24, 25, 29]. None of the larger studies showed the remarkable treatment response seen in the case reports and in the one patient in our study. However, the larger studies were all done for a short period of time, with less rigorous KD, with less fat and more carbohydrate content. Louw et al. used 4:1 liquid KD but only for 8 weeks, after which the diet was switched to 1.5–2:1 ratio [30] in which only ~ 60–75% of calories are derived from fat. In experimental setting, efficacy is linked to the more restricted diets such as the 4:1 diet. Even with such diets, however, glucose deprivation and ketone body exposure by themselves only retard proliferation and slows tumor growth in animals but do not kill all tumor cells [16]. It is therefore likely that a short KD course may at best retard GBM growth with re-growth following KD withdrawal. Longer treatment duration, e.g., 1 year or longer, may be needed. There is at present little pre-clinical or clinical data to indicate how long KD treatment should last. We treated patients for 6 months, which is longer by almost 3 months than any previous study other than case reports [26–29]. Four of our subjects continued KD on their own past the protocol duration, including one each for total of 26 and 19 months. This was relatively easy because during the 6 months of TMR they learned the meals to cook. In epilepsy, 4:1 and 3:1 KD with CH of 10–20 g/day is sometimes continued for many years [33–35]. Our study shows such treatment is feasible in GBM patients also when TMR program is used.

### Timing of intervention and its potential impact on efficacy

All our newly diagnosed patients started KD after surgical resection, in conjunction with initiation of radiation and temozolomide therapy. There was an approximately 6–8 weeks lag between diagnosis and KD initiation. It has been suggested that KD should be started before surgical resection and before radiation and temozolomide to allow maximal impact of KD [14]. This did not occur in our patients. It is possible that the time lag may have impacted KD efficacy [29]. The most remarkable animal study of KD in malignant glioma showed cure in animals treated with 4:1 KD concurrently with radiation treatment [22]. This was done by Louw et al. who started 4:1 KD 2 weeks before radiation and chemotherapy, albeit after surgery, continued it for 8 weeks, and then switched to 1.2–2:1 KD for further 6 weeks. Six of 9 patients completed the protocol, and 4 patients continued KD after the 14 weeks of study treatment. The median survival of 12.8 months was unremarkable [30]. Future studies may, therefore, consider study starting KD before surgery [29].

### Limitations

The study has significant limitations. It is very small, has two different GBM patient populations (newly diagnosed and recurrent), is open label, and had potential for patient self-selection bias. KD is not a recognized treatment for GBM. Participants were all patients who were seeking additional treatment options and were highly motivated. Concomitant treatments were allowed. The two subjects with longer than expected survival used other therapies (bevacizumab, with pre-KD progression, but continued with KD and BCNU, started 4 months after KD in one patient, and various supplements in the other patient). Factors that may affect survival such as age, genetic background, and concomitant treatments were not controlled. The TMR was “one size fits all,” without caloric adjustment for gender, size, or activity. We are adapting the program to allow such flexibility in future.

### Recruitment

In addition, we failed to recruit the planned number of subjects. During almost 5 years of recruitment, only 8 subjects were recruited. A much larger number of eligible patients were screened, with eligible screened: enrolled patient ratio 3.4:1. Reasons for non-enrollment varied. The largest was discouragement by treating oncologists and competing studies by neurosurgeons. We made a large recruitment education effort targeted at local oncologists and neurosurgeons, with little success and only two referrals. The second largest reason was patients’ unwillingness to restrict the pleasures of food and alcohol during the perceived short remainder of their life. This is a major limitation. It may not change unless and until KD treatment is shown to improve outcome.

### Caloric restriction

Animal evidence suggests that caloric restriction may have an additional benefit in KD [7, 8]. This has led to proposals that KD in GBM be calorically restricted, for example to 600–1200 kcal/day, supported by a couple of remarkable case reports [25, 29]. Our study shows that caloric restriction may not be feasible, except in exceptional individuals. Our diet was only modestly calorically restricted at 1600 kcal/day, and yet it was associated with weight loss (and hunger) in all subjects treated for > 1 month. This was unacceptable to patients, families, and treating oncologists, leading to caloric increase to prevent further weight loss.

### Steroid reduction

Steroids are used routinely in GBM for treatment of cerebral edema. They elevate blood glucose. Hyperglycemia is associated with reduced survival in GBM patients [23] and in animals [17], and with worse functional outcome after surgical resection in GBM patients [36]. We were able to withdraw (*n* = 2) or reduce (*n* = 1) the dose of dexamethasone in 3/5 patients who were taking it before KD. In two, there was no edema during the ensuing radiation and temozolomide treatment. In another, prominent edema resolved after KD initiation even as the dexamethasone dose was tapered. This has been reported previously [25, 28, 29]. KD reduces vascular permeability and edema in a mouse glioma model [9]. Substitution of steroid therapy for edema with KD and the associated avoidance of steroid-induced hyperglycemia may provide benefit independent of KD effect on the tumor cells and should be further evaluated.

### Compliance

Compliance is an issue with any diet, more so in patients with terminal illness. However, our study suggests that complete ready-made cooked meal replacement may be conducive to compliance: 4/7 patients treated for > 1 week had complete compliance, 2 had substantial compliance, and only 1 had poor compliance. Patients with recurrent GBM (group 2) had poorer compliance. This was reflected in lower ketone levels.

### “Lessons learned” for future studies

The major limitation of the study was failure to recruit. For future studies to succeed, this barrier has to be overcome. There were two major reasons: (1) lack of acceptance of the KD cancer treatment concept by oncologists. Future studies should strive to be done in close collaboration with neuro-oncologists and oncologists, preferably in academic oncology centers and in international collaborations, with full participation by the oncologists. For this to happen, the ketogenic community need to educate the oncology community about the validity of the basic science of dietary cancer and GBM treatment to justify human intervention studies. (2) During our study, KD was unknown, thought of as “weird,” and patients were mistrustful of it. Recruitment will be easier if the diet is accepted by the broad public. Fortunately, there has been a dramatic rise in the popularity of ketogenic diets in the last 2 years. This offers an opportunity for a new effort. Additional future improvements may include avoidance of caloric restriction and greater flexibility of patient choice of the meals from the available menu, to be tailored a la carte by each patient from the menu of available meals. This can be done with an app which we developed, but did not activate during the study because of the cost.

## Conclusions

This pilot study shows that long-term rigorous ketogenic diet treatment of patients with malignant cancer such as GBM is well tolerated in long-term treatment with the use of a standardized total meal replacement program using ready-made cooked meals. If issues with recruitment can be resolved, this approach allows testing of KD in GBM and other malignant cancers in a standardized way on large cohorts of patients.

## Data Availability

The datasets used during the current study are available from the corresponding author on a reasonable request.

## Abbreviations

BHB: Beta-hydroxy butyrate
BCNU: Bis-chloroethylnitrosourea
BMI: Body mass index
CH: Carbohydrate
CBC: Complete blood count
FPG: Fasting blood glucose
GBM: Glioblastoma
HDL: High density lipoprotein
IDH: Isocitrate dehydrogenase
KD: Ketogenic diet
LDL: Low density lipoprotein
MRI: Magnetic resonance imaging
MGMT: O6-methylguanine methyltransferase promoter methylation
SAE: Serious adverse event
TMR: Total meal replacement
TEAEs: Treatment-emergent adverse events (TEAEs)

## References

[1] Ostrom QT, Bauchet L, Davis FG, Deltour I, Fisher JL, Langer CE (2014). The epidemiology of glioma in adults: a “state of the science” review. Neuro Oncol.

[2] Gittleman H, Boscia A, Ostrom QT, Truitt G, Fritz Y, Kruchko C, et al. Survivorship in adults with malignant brain and other central nervous system tumor from 2000-2014. Neuro Oncol. 2018; 10.1093/neuonc/noy090. 10.1093/neuonc/noy090 PMC6225751 29850889

[3] Stupp R, Mason WP, van den Bent MJ, Weller M, Fisher B, Taphoorn MJB (2005). Radiotherapy plus concomitant and adjuvant temozolomide for glioblastoma. N Engl J Med.

[4] Jeswani S, Nuño M, Folkerts V, Mukherjee D, Black KL, Patil CG. Comparison of survival between cerebellar and supratentorial glioblastoma patients: surveillance, epidemiology, and end results (SEER) analysis. Neurosurgery. 2013;73:240–6; discussion 246; quiz 246. 10.1227/01.neu.0000430288.85680.37. 10.1227/01.neu.0000430288.85680.37 23615082

[5] Clarke J, Butowski N, Chang S (2010). Recent advances in therapy for glioblastoma. Arch Neurol.

[6] Champ CE, Palmer JD, Volek JS, Werner-Wasik M, Andrews DW, Evans JJ (2014). Targeting metabolism with a ketogenic diet during the treatment of glioblastoma multiforme. J Neurooncol.

[7] Maroon JC, Seyfried TN, Donohue JP, Bost J (2015). The role of metabolic therapy in treating glioblastoma multiforme. Surg Neurol Int.

[8] Seyfried TN, Flores R, Poff AM, D’Agostino DP, Mukherjee P (2015). Metabolic therapy: a new paradigm for managing malignant brain cancer. Cancer Lett.

[9] Woolf EC, Syed N, Scheck AC. Tumor metabolism, the ketogenic diet and β-hydroxybutyrate: novel approaches to adjuvant brain tumor therapy. Front Mol Neurosci. 2016; 10.3389/fnmol.2016.00122. 10.3389/fnmol.2016.00122 PMC5110522 27899882

[10] Schwartz L, Seyfried T, Alfarouk KO, Da Veiga Moreira J, Fais S (2017). Out of Warburg effect: an effective cancer treatment targeting the tumor specific metabolism and dysregulated pH. Semin Cancer Biol.

[11] Winter SF, Loebel F, Dietrich J (2017). Role of ketogenic metabolic therapy in malignant glioma: a systematic review. Crit Rev Oncol Hematol.

[12] Noorlag L, De Vos FY, Kok A, Broekman MLD, Seute T, Robe PA (2019). Treatment of malignant gliomas with ketogenic or caloric restricted diets: a systematic review of preclinical and early clinical studies. Clin Nutr.

[13] Seyfried TN, Shelton L, Arismendi-Morillo G, Kalamian M, Elsakka A, Maroon J, et al. Provocative question: should ketogenic metabolic therapy become the standard of care for glioblastoma? Neurochem Res. 2019; 10.1007/s11064-019-02795-4. 10.1007/s11064-019-02795-4 31025151

[14] Seyfried TN, Kiebish MA, Marsh J, Shelton LM, Huysentruyt LC, Mukherjee P (2011). Metabolic management of brain cancer. Biochim Biophys Acta.

[15] Zhou W, Mukherjee P, Kiebish MA, Markis WT, Mantis JG, Seyfried TN. The calorically restricted ketogenic diet, an effective alternative therapy for malignant brain cancer. Nutr Metab (Lond). 2007;4(5) 10.1186/1743-7075-4-5. 10.1186/1743-7075-4-5 PMC1819381 17313687

[16] Martuscello RT, Vedam-Mai V, McCarthy DJ, Schmoll ME, Jundi MA, Louviere CD (2016). A supplemented high-fat low-carbohydrate diet for the treatment of glioblastoma. Clin Cancer Res.

[17] Seyfried TN, Marsh J, Shelton LM, Huysentruyt LC, Mukherjee P (2012). Is the restricted ketogenic diet a viable alternative to the standard of care for managing malignant brain cancer?. Epilepsy Res.

[18] Mukherjee P, Abate LE, Seyfried TN (2004). Antiangiogenic and proapoptotic effects of dietary restriction on experimental mouse and human brain tumors. Clin Cancer Res.

[19] Seyfried TN, Mukherjee P. Targeting energy metabolism in brain cancer: review and hypothesis. Nutr Metab (Lond). 2005;2(30) 10.1186/1743-7075-2-30. 10.1186/1743-7075-2-30 PMC1276814 16242042

[20] Augur ZM, Doyle CM, Li M, Mukherjee P, Seyfried TN (2018). Nontoxic targeting of energy metabolism in preclinical VM-M3 experimental glioblastoma. Front Nutr.

[21] Mukherjee P, Augur ZM, Li M, Hill C, Greenwood B, Domin MA (2019). Therapeutic benefit of combining calorie-restricted ketogenic diet and glutamine targeting in late-stage experimental glioblastoma. Commun Biol.

[22] Abdelwahab MG, Fenton KE, Preul MC, Rho JM, Lynch A, Stafford P (2012). The ketogenic diet is an effective adjuvant to radiation therapy for the treatment of malignant glioma. PLoS One.

[23] Derr RL, Ye X, Islas MU, Desideri S, Saudek CD, Grossman SA (2009). Association between hyperglycemia and survival in patients with newly diagnosed glioblastoma. J Clin Oncol.

[24] Nebeling LC, Miraldi F, Shurin SB, Lerner E (1995). Effects of a ketogenic diet on tumor metabolism and nutritional status in pediatric oncology patients: two case reports. J Am Coll Nutr.

[25] Maroon J, Bost J, Amos A, Zuccoli G (2013). Restricted calorie ketogenic diet for the treatment of glioblastoma multiforme. J Child Neurol.

[26] Rieger J, Bähr O, Maurer GD, Hattingen E, Franz K, Brucker D (2014). ERGO: a pilot study of ketogenic diet in recurrent glioblastoma. Int J Oncol.

[27] Schwartz KA, Noel M, Nikolai M, Chang HT (2018). Investigating the ketogenic diet as treatment for primary aggressive brain cancer: challenges and lessons learned. Front Nutr.

[28] Santos JG, Da Cruz WMS, Schönthal AH, Salazar MD, Fontes CAP, Quirico-Santos T (2018). Efficacy of a ketogenic diet with concomitant intranasal perillyl alcohol as a novel strategy for the therapy of recurrent glioblastoma. Oncol Lett.

[29] Elsakka AMA, Bary MA, Abdelzaher E, Elnaggar M, Kalamian M, Mukherjee P (2018). Management of glioblastoma multiforme in a patient treated with ketogenic metabolic therapy and modified standard of care: a 24-month follow-up. Front Nutr.

[30] van der Louw EJTM, Olieman JF, van den Bemt PMLA, Bromberg JEC, Oomen-de Hoop E, Neuteboom RF, et al. Ketogenic diet treatment as adjuvant to standard treatment of glioblastoma multiforme: a feasibility and safety study. Ther Adv Med Oncol 2019;11:1758835919853958. 10.1177/1758835919853958. 10.1177/1758835919853958 PMC6589986 31258628

[31] Martin-McGill KJ, Srikandarajah N, Marson AG, Tudur Smith C, Jenkinson MD (2018). The role of ketogenic diets in the therapeutic management of adult and paediatric gliomas: a systematic review. CNS Oncol.

[32] Schmidt M, Pfetzer N, Schwab M, Strauss I, Kämmerer U (2011). Effects of a ketogenic diet on the quality of life in 16 patients with advanced cancer: a pilot trial. Nutr Metab (Lond).

[33] Kossoff EH, Zupec-Kania BA, Amark PE, Ballaban-Gil KR, Christina Bergqvist AG, Blackford R (2009). Optimal clinical management of children receiving the ketogenic diet: recommendations of the International Ketogenic Diet Study Group. Epilepsia.

[34] Vining EPG (2008). Long-term health consequences of epilepsy diet treatments. Epilepsia.

[35] Klein P, Tyrlikova I, Mathews GC (2014). Dietary treatment in adults with refractory epilepsy: a review. Neurology.

[36] Link TW, Woodworth GF, Chaichana KL, Grossman SA, Mayer RS, Brem H (2012). Hyperglycemia is independently associated with post-operative function loss in patients with primary eloquent glioblastoma. J Clin Neurosci.

